# Iptakalim, a novel ATP-sensitive potassium channel opener, inhibits pulmonary arterial smooth muscle cell proliferation by downregulation of PKC-α^☆^

**DOI:** 10.1016/S1674-8301(11)60052-3

**Published:** 2011-11

**Authors:** Xiangrong Zuo, Feng Zong, Hui Wang, Qiang Wang, Weiping Xie, Hong Wang

**Affiliations:** a Department of Respiratory Medicine;; b Critical Care Medicine, the First Affiliated Hospital of Nanjing Medical University, Nanjing, Jiangsu 210029, China;; c Department of Respiratory Medicine, No. 81 Hospital of PLA, Nanjing, Jiangsu 210002, China.

**Keywords:** iptakalim, pulmonary arterial smooth muscle cells (PASMCs), pulmonary hypertension, protein kinase C-α (PKC-α), hypoxia, proliferation

## Abstract

Iptakalim is a new ATP-sensitive potassium (K_ATP_) channel opener, and it inhibits the proliferation of pulmonary arterial smooth muscle cells (PASMCs) and pulmonary vascular remodeling. However, the underlying mechanism remains unclear. In the present study, we found that iptakalim significantly decreased pulmonary artery pressure, inhibited pulmonary ariery remodeling and PKC-α overexpression in chronic hypoxia in a rat pulmonary hypertension model. Iptakalim reduced hypoxia-induced expression of PKC-α, and abolished the effect of hypoxia on PASMC proliferation significantly in a dose-dependent manner *in vitro*. Moreover, these effects were abolished by glibenclamide, a selective K_ATP_ channel antagonist. These results indicate that iptakalim inhibits PASMC proliferation and pulmonary vascular remodeling induced by hypoxia through downregulating the expression of PKC-α. Iptakalim can serve as a novel promising treatment for hypoxic pulmonary hypertension.

## INTRODUCTION

Hypoxia-induced pulmonary hypertension is a common complication of residence at high altitudes and chronic lung diseases such as chronic obstructive pulmonary disease (COPD), various interstitial lung diseases, bronchiectasis, asthma, and sleep apnea[1],[2]. It is widely accepted that hypoxic pulmonary hypertension is strongly associated with increased morbidity and reduced survival[3]. Hypoxic pulmonary vascular structural remodeling is the key pathological basis of chronic hypoxic pulmonary hypertension[4],[5], although the underlying mechanism is not fully understood. Numerous studies have demonstrated that hypoxia-induced abnormal proliferation of pulmonary arterial smooth muscle cells (PASMCs) may be a key component of pulmonary vascular remodeling[6]–[9].

The protein kinase C (PKC) is one component of the crucial intracellular signal transduction systems and a key enzyme in the regulation of cell permeability, contraction, proliferation, differentiation, apoptosis, and secretion[10]. There are at least 12 distinct isoforms in the PKC family. Among them, PKC-α is a conventional isoform, which requires calcium, phosphatidylserine, and diacylglycerol (DAG), or phorbol esters for full activation. PKC-α is widely expressed and plays important roles in several cellular processes and pathologies, involving cancer, cardiovascular disorders, atherogenesis, thrombosis and pulmonary disease. Therefore, selectively targeted modulation of PKC-α has potential therapeutic value in the future treatment and management of these diseases[11]. PKC-α is also found to be distributed in the cytosol of PASMCs[12]. Previous works suggested that PKC-α was closely associated with the abnormal proliferation of PASMCs and pulmonary vascular remodeling induced by cigarette smoking[13] or hypoxia[14],[15]. The expression of PKC-α was also significantly increased in the lung tissues from idiopathic pulmonary arterial hypertension patients[16].

Potassium channel dysfunction is evident in pulmonary hypertension. Potassium channels contribute to the regulation of pulmonary vascular tone and vascular remodeling through their effects on cytoplasmic K^+^ and Ca^2+^ concentrations and membrane potential, thereby regulating cell migration, proliferation, and apoptosis[17]. There are at least four types of potassium channels. Among them, ATP-sensitive potassium (K_ATP_) channels belong to the Kir (inward rectifier) superfamily of potassium channels, and have received intense scrutiny for their biophysical properties, regulation, and pharmacology during the past two decades[18]. K_ATP_ channels are widely expressed in the cardiovascular system including the pulmonary circulation. K_ATP_ channels contribute to the maintenance of membrane potential (Em) in PASMCs. Closure of K_ATP_ channels could lead to increased [Ca^2+^]_i_ and enhanced contraction and proliferation of PASMCs. Opening of K_ATP_ channels causes an increase of K^+^ efflux, membrane hyperpolarization, inhibition of Ca^2+^ influx, and subsequent vascular smooth muscle relaxation[19]. K_ATP_ channels appears suppressed in proliferating PASMCs[20]. Therefore, pharmacological manipulation of K_ATP_ channels in pulmonary vascular cells represents a potential therapeutic target in the treatment of pulmonary hypertension[21].

Iptakalim, a fatty para-amino compound with low molecular weight, has been confirmed by substantial pharmacological, biochemical, and electrophysiological studies, as well as receptor-combining tests as a selective K_ATP_ channel opener[22]. Our previous studies have revealed that iptakalim could alleviate pulmonary artery remodeling and pulmonary hypertension in chronic hypoxic rats and inhibit the proliferation of rabbit or human PASMCs induced by endothelin-1 (ET-1)[23]–[25].

However, the underlying mechanisms are not fully understood. It is known that overexpression of PKC-α in pulmonary arterioles is involved in the development of pulmonary vascular remodeling in pulmonary hypertension[13],[26] and the K_ATP_ channel opener cromakalim attenuated the pulmonary vasoconstrictory effect of PKC activation[27]. Therefore, in the present study, we used a rat chronic hypoxia exposure model to study whether iptakalim could inhibit the proliferation of PASMCs and pulmonary vascular remodeling by regulating the expression of PKC-α in PASMCs.

## MATERIALS AND METHODS

### Animals and administration

Sixty young male Sprague-Dawley rats (180-220 g) were obtained from Shanghai Laboratory Animal Center, Chinese Academy of Sciences (Certificate No. SCXK 2007-0005, Grade II) and allowed 1 week to acclimate. All rats were maintained on a 12 h light-dark cycle (light on from 8:00 to 20:00) at ambient temperature (22°C-24°C) with free access to standard rodent chow and tap water. All animal works were approved by the Animal Care and Use Committee of Nanjing Medical University.

Iptakalim was synthesized and provided by the Institute of Pharmacology and Toxicology, Academy of Military Medical Sciences, China, and was dissolved in 0.9% saline to make a concentration of 0.30 g/mL for oral use. Pulmonary hypertension was induced by exposure to hypoxia (10% inspired O_2_ fraction) in a normobaric chamber as described previously[23]. Forty-five rats were placed into this hypoxia chamber for 8 h/d, 6 d/week for 4 weeks. Three subgroups of rats were treated with either iptakalim at a dose of 0.75 or 1.5 mg/(kg • d) or with vehicle alone (0.9% saline) by gavage tube, once a day, before the rats were exposed to the same hypoxic condition (*n* = 15, per group), while age- and weight-matched control animals were kept at normobaric pressure (760 mmHg) and room air (21%O_2_).

### Measurement of mean pulmonary artery pressure

Mean pulmonary artery pressure (mPAP) was measured by the method as we previously described[23]. Briefly, after 4 weeks of hypoxia, all rats were intraperitoneally anesthetized with urethane (1.0 g/kg) and placed on the table. A small polyethylene catheter (PE10, Becton Dickinson, Frankin Lakes, NJ, USA) was advanced from the right jugular vein into the right ventricle (RV) and the main pulmonary artery under the guidance of the pressure tracing. After 20 min stabilization, mPAP was recorded using a miniature pressure transducer (TSD104A, BIOPAC Systems., Santa Barbara, CA, USA), digitized by a BIOPAC MP100 data acquisition system, and stored in the computer. After the completion of haemodynamic measurements, a thoracotomy was performed. The whole lungs and heart were rapidly removed and flushed with ice-cold normal saline to remove any blood.

### Assessment of right ventricular hypertrophy

During harvest, the atria, the pulmonary trunk, and the aorta were removed from the heart. The right ventricular wall was separated from the left ventricular wall and ventricular septum. Wet weights of the right ventricle, free left ventricular wall, and ventricular septum were determined. Right ventricular hypertrophy was expressed as the ratio of weight of the right ventricular wall (RV) to that of the free left ventricular wall and ventricular septum (LV+S).

### Morphometric analysis of the pulmonary arteries

The left lungs of the control, hypoxia and iptakalim treatment groups were cut and fixed with 4% phosphate-buffered formalin. Then, the lung specimens were embedded in paraffin, cut into 4-µm-thick sections and subjected to hematoxylin-eosin (H&E) and Weigert elastic staining before light microscopy. Medial thickness (MT) and external diameter (ED) were measured from 8 to 10 arterioles per animal using Image Pro-Plus 6.0 software (Media Cybernetics, USA) for assessing pulmonary vascular remodeling. ED was defined as distance between the external elastic laminae, while MT was determined as distance between the external and internal elastic laminae. The percentage of MT was calculated using the following formula: MT (%) = (2×MT/ED) × 100%.

### Immunohistochemical staining

Immunohistochemical staining was performed by using the EnVision two-step detection method (EnVision Detection Kit, Gene Tech Co., Ltd, Shanghai, China), and the operations were carried out strictly following the manufacturer's instructions. In brief, the deparaffinized formalin-fixed sections were placed in EDTA retrieval agent at pH8.0, and autoclaved at 121°C for 20 min for antigen retrieval. The sections were washed in phosphate-buffered saline (PBS, pH7.6), and incubated overnight at 4°C with either a rabbit anti-PKC-α polyclonal antibody (1:100, Bioworld Technology, Inc., Minneapolis, MN, USA) or a mouse anti-PCNA primary antibody (1:50, Maxim Biotect, Fuzhou, China). After the sections were washed with PBS three times for 5 min, they were treated for 30 min at room temperature in ChemMate ™EnVision+/HRP. Subsequently, the sections were washed with PBS, and diaminobezidine (DAB) colorization was applied. Staining was monitored under a brightfield microscope, and slices were then counterstained with hematoxylin. Negative controls for each tissue section were prepared by omitting the primary antibody. The activity of PKC-α in arterioles was quantified by measuring the mean optical density (OD) using Image Pro-Plus 6.0 software. In each slide, 6 randomly selected muscular arterioles (diameter≤200 µm) were measured and the average was calculated as the representative value for each rat. The PCNA index, calculated as the number of cells staining positive divided by the total number of PASMCs in the muscular arterioles, was used as an indicator of PASMC proliferation.

### Human pulmonary arterial smooth muscle cell (PASMC) culture and treatment

Primary PASMCs were isolated from human pulmonary arteries and cultured as we previously reported[25]. Briefly, after approval by the Ethical Review Board of the First Affiliated Hospital of Nanjing Medical University, we obtained specimens of human pulmonary arteries from the healthy segments of the lung of patients undergoing pulmonary resection. After the intrapulmonary arteries (3rd-4th division) were excised from the adventitia and opened longitudinally, the endothelial cells of the pulmonary arteries were removed mechanically with a scalpel blade, and the vessels were cut into 1-3 mm^2^ pieces. Pieces of the arteries were incubated at 37°C under humidified normoxia (21% O_2_, 5% CO_2_ and 74% N_2_) in the Medium 231(Cascade Biologics, Inc., Portlant, OR, USA) containing 20% fetal bovine serum (FBS), 100 µg/mL streptomycin, 100 U/mL penicillin, 2.5 µg/mL amphotericin B. Cells were confluent after 3-4 weeks, then digested with 0.25% trypsin and subcultured. Cells from passage 4 and 6 were used for the following experiments. Human PASMCs were identified by characteristic “hill-and-valley” morphology and the positive expression for α-smooth muscle actin demonstrated by immunocytochemical staining in more than 95% of cells.

Human PASMCs were suspended in M231 containing 10% FBS, seeded in 96-well plates (1×10^4^ cells per well) or T25 flask, grown until 80% to 90% confluent, and brought to quiescence by incubation with serum-free M231 for 24 h. Then, the cells were cultured in normoxia or hypoxia (5% O_2_, 5% CO_2_, and 90% N_2_) for 24 h. To determine whether hypoxia-induced human PASMC proliferation and PKC-α activation could be inhibited by iptakalim, the cells were pretreated with iptakalim (0, 0.1, 1.0, and 10 µmol/L, respectively) for 30 min prior to treatment with hypoxia. To determine whether the effects of iptakalim on hypoxia-induced human PASMC proliferation and PKC-α activation could be blocked by K_ATP_ channel antagonist, human PASMCs were preincubated with 0.1 or 1.0 or 10 µmol/L glibenclamide, a selective K_ATP_ channel antagonist, for 30 min prior to treatment with 10 µmol/L iptakalim and 24 h hypoxia.

### [^3^H] thymidine incorporation assay for determination of cell proliferation

Agents were added into fresh M231 as indicated above and cells were incubated with this test substance for 24 h in normoxia or hypoxia as described above. [^3^H]Thymidine was added to each well at a final concentration of 0.5 Ci/well 6 h before termination of the cultures. At the end of the incubation period, the medium was removed, and the cell monolayer was washed with ice-cold PBS and digested with trypsin. DNA incorporated with [^3^H]thymidine was precipitated in cold 7.5% trichloroacetic acid, centrifuged, washed three times with 7.5% trichloroacetic acid, and then counted on a LKB Rackbeta scintillation counter. Experiments were performed for six times independently in duplicates.

### Western blotting analysis

Protein was extracted from both pulmonary arterial tissues and cell lysates and the concentration of protein was determined by the BCA method. Protein samples were loaded in each lane and subjected to SDS-PAGE gel (10%) for separation. The PVDF membrane was used for immunoblotting. The membrane was blocked and incubated with antibodies against PKC-α (Bioworld, USA) or against GAPDH (KangChen Bio-tech Inc, Shanghai, China) overnight at 4°C. After washing with TBST, the membrane was incubated for 1 h with HRP-labeled anti-mouse IgG. The immunoreactive bands were visualized with enhanced chemiluminescence and captured on X-ray film. The optical density of the bands was measured with a gel imaging analysis system. The results were expressed as the ratio of the expression of PKC-α to that of GAPDH.

### Statistical analysis

The results are expressed as mean±SD. Statistical analysis was performed using the unpaired Student's *t*-test or ANOVA and post hoc tests (Student–Newman–Keuls) as indicated. A value of *P* < 0.05 was considered significant.

## RESULTS

### Measurement of mean pulmonary artery pressure

After exposure to hypoxia for 4 weeks, mPAP in the hypoxia group was significantly increased by 69.95% compared with that of control group (*P* < 0.05). Treatment of rats with 0.75 and 1.5 mg/(kg • d) iptakalim decreased mPAP by 32.63% and 37.20% of the untreated hypoxia group, respectively (***Table 1***).

**Table 1:** Comparisons of mPAP and RV/(LV+S) in the four groups

Group	mPAP (mmHg)	RV/(LV+S)
Control	18.90±1.30	0.26±0.02
Hypoxia	32.12±3.77*	0.39±0.04*
Ipt treatment [0.75 mg/(kg·d)]	21.64±2.29^#^	0.29±0.02^#^
Ipt treatment [1.50 mg/(kg·d)]	20.17±2.19^#^	0.27±0.02^#^

mPAP: mean pulmonary artery pressure, RV/(LV+S): right ventricular weight / left ventricular weight plus septal weight. Ipt: iptakalim. Data are mean±SD, *n* = 15. *P < 0.05 *vs* control; ^#^*P* < 0.05 *vs* hypoxia.

### Assessment of right ventricular hypertrophy

The animals subjected to chronic hypoxia for 4 weeks exhibited the expected right heart hypertrophy, the RV/(LV+S) ratio in the hypoxia group was increased by 32.42%, compared to the control group. Pretreatment with iptakalim significantly decreased the RV/(LV+S) ratio by 15.93% and 19.15%, respectively, as compared to the untreated hypoxia group (*P* < 0.05, ***Table 1***).

### Histology of the lungs of rats with pulmonary hypertension

**Fig.1:**
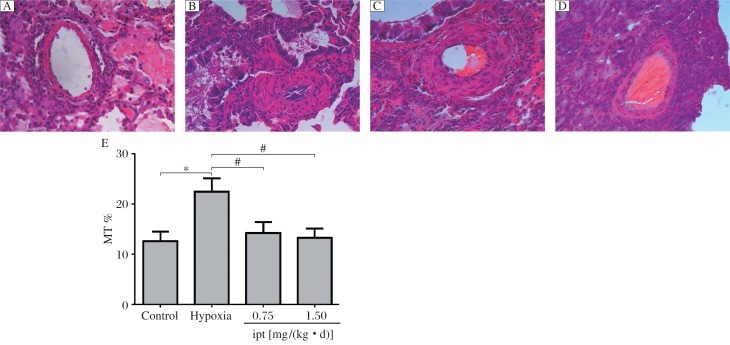
Effects of iptakalim on lung vascular morphology in hypoxia-induced pulmonary hypertension rats (Magnification ×400). Lung and pulmonary arterioles were stained with hematoxylin and eosin (H&E). A: the control group. B: the hypoxia group. C: the iptakalim treatment group [0.75 mg/(kg • d)]. D: the iptakalim treatment group [1.50 mg/(kg • d)]. E: effects of iptakalim on the percentage of medial thickness (MT) in pulmonary arterioles of rats with chronic hypoxia-induced pulmonary hypertension. *P < 0.05, Ipt: iptakalim.

**Fig. 2:**
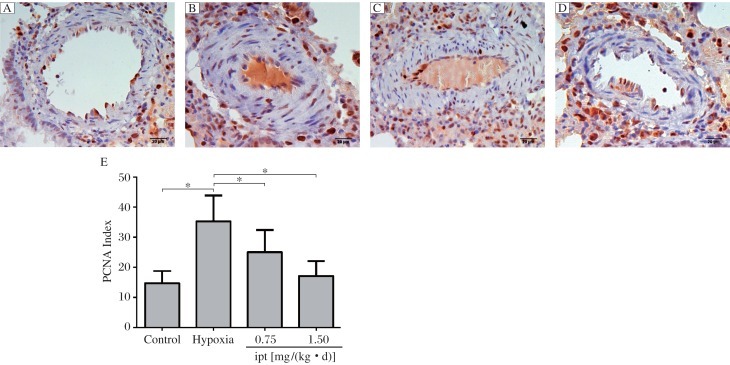
Immunohistochemistry for proliferative cell nuclear antigen (PCNA) in rat lungs (Magnification ×400). A: the control group, B: the hypoxia group, C: the iptakalim treatment group [0.75 mg/(kg • d)], D: the iptakalim treatment group [1.50 mg/(kg • d)]. E: Effects of iptakalim on the proliferation index in pulmonary arteries of rats with chronic hypoxic pulmonary hypertension. *P < 0.05. Ipt: iptakalim.

Morphometric analysis revealed that chronic hypoxia resulted in structural changes of small intrapulmonary arteries in rat lungs. These changes were mainly characterized by thickening of the medial layer (proliferation of vascular smooth muscle cells, ***Fig. 1A*** and ***Fig. 1B***) and significant increase in the percentage of MT (%), (22.47±2.63)% *vs* (12.61±1.86)% for control (***Fig. 1E***). While the medial muscle hypertrophy between the external and the internal elastic lamina in iptakalim [0.75 and 1.5 mg/(kg • d), i.g.] treatment group were attenuated compared with the hypoxia group, the medial layer was close to that of the control group (***Fig. 1C***, ***Fig. 1D***, and ***Fig. 1E***). In hypoxic rats, PCNA labeling showed proliferation of smooth muscle cells in distal pulmonary vessel walls (***Fig. 2B***). PCNA-positive cells were (35.25±8.63)% in the muscularized pulmonary vessels of the hypoxia group compared with the control group (14.69±4.20)% (***Fig. 2E***). Compared to the hypoxia group of (35.25±8.63)%, rats treated with iptakalim [0.75 and 1.5 mg/(kg • d), i.g.] after 4 weeks also had a significant decrease in pulmonary artery PCNA index, (25.05±7.40)% *vs.* (17.09±4.98)%, respectively (***Fig. 2C***, ***Fig. 2D*** and ***Fig. 2E***). These results indicate that iptakalim suppressed PASMC proliferation and attenuated pulmonary artery remodeling induced by hypoxia.

### Effect of iptakalim on PKC-α expression in the lungs

**Fig. 3:**
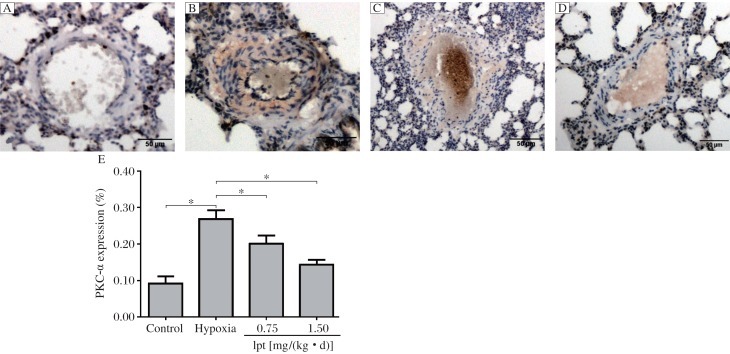
The expression of PKC-α in the lungs was detected by immunohistochemical staining (Magnification: ×200). A: the control group. B: the hypoxia group. C: the iptakalim treatment group [0.75 mg/(kg • d)], D: the iptakalim treatment group [1.5 mg/(kg • d)]. E: effects of iptakalim on PKC-α expression (represented by the mean percentage changes in mean optical intensity) in the vessel wall of pulmonary arterioles of rats with chronic hypoxia-induced pulmonary hypertension. *P < 0.05. Ipt: iptakalim.

**Fig. 4:**
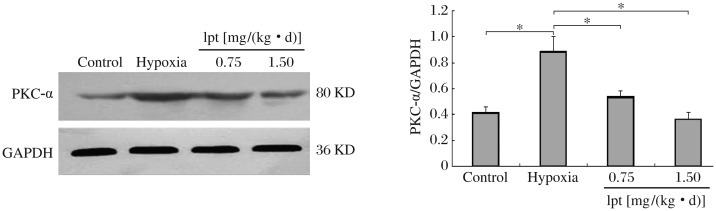
Effects of iptakalim on the expression of PKC-α in the pulmonary arteries of rats with chronic hypoxia induced pulmonary hypertension. *P < 0.05. lpt: iptakalim.

The expression of PKC-α protein in the lungs was detected by immunohistochemical staining and Western blot analysis. Immunohistochemical analysis showed that PKC-α was expressed weakly in the cytoplasm of smooth muscle cells in the medial layers of muscular arterioles in the control group. In contrast, the medial smooth muscule was strongly positive for PKC-α after exposure to hypoxia (***Fig. 3***). As shown in ***Fig. 3E***, PKC-α expression, as the mean percentage changes in mean optical intensity, in rats kept in hypoxia increased significantly, compared with that in the control group after 4 weeks (*P* < 0.05). Immunohistochemical analysis showed that the expression of PKC-α was significantly decreased in PASMCs of the iptakalim treatment group [0.75 and 1.5 mg/(kg • d)] compared with those of the hypoxia group (*P* < 0.05). Similarly, Western blotting of the lung tissues showed an increase in the expression of PKC-α in the hypoxia group compared with the control group (*P* < 0.05, ***Fig. 4A***). Iptakalim reversed hypoxia-induced increase in PKC-α expression in a dose-dependent manner (*P* < 0.05, ***Fig. 4B***).

### Iptakalim inhibits hypoxia-induced proliferation of human PASMCs by opening K_ATP_ channel

**Fig. 5:**
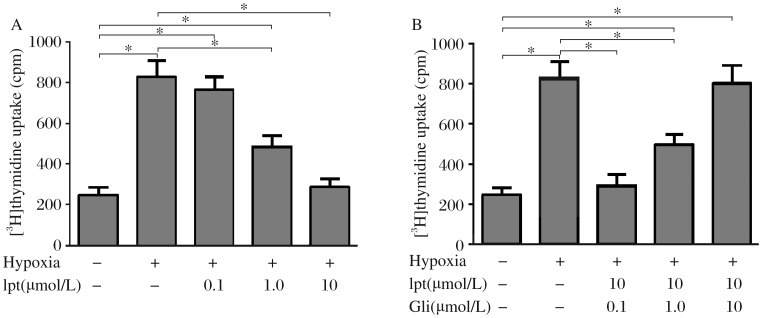
Effect of iptakalim (Ipt) with or without glibenclamide (Gli) on hypoxia-induced proliferation of human PASMCs. A: The human PASMCs were treated with hypoxia for 24 h with or without iptakalim at 0.1-10 µmol/L and iptakalim dose-dependently inhibited the proliferation of human PASMCs induced by hypoxia. B: Pretreatment with glibenclamide at 0.1-10 µmol/L 30 min before Ipt treatment (10 µmol/L) and hypoxia dose-dependently blocked the anti-proliferation effect of iptakalim. The proliferation of human PASMCs was determined by [^3^H]thymidine incorporation assay. *P < 0.05, *n* = 6.

As shown in ***Fig. 5***, [^3^H]thymidine incorporation was increased 3.38 folds in cells treated with hypoxia compared to cells in normoxia. When the cells were treated with 0.1, 1.0 or 10 µmol/L iptakalim, [^3^H]thymidine incorporation was decreased by 7.40%, 41.26% and 65.28%, respectively, compared with those treated with hypoxia alone. Consequently, iptakalim inhibited the proliferation of human PASMCs induced by hypoxia in a dose-dependent manner. When cells were pre-incubated with 0.1, 1.0 or 10 µmol/L glibenclamide for 30 min prior to the addition of 10 µmol/L iptakalim and 24 h hypoxia treatment, [^3^H]thymidine uptake was increased by 21.41%, 103.93%, 228.52% and 263.57%, respectively, compared to normoxic conditions. Consequently, glibenclamide abolished the inhibition of iptakalim on hypoxia-induced proliferation of human PASMCs in a dose-dependent manner. These results showed that iptakalim almost completely inhibited the proliferation of PASMCs induced by hypoxia by opening K_ATP_ channel.

### Iptakalim downregulates the expression of PKC-α in human PASMCs under hypoxia by opening K_ATP_ channel

**Fig. 6:**
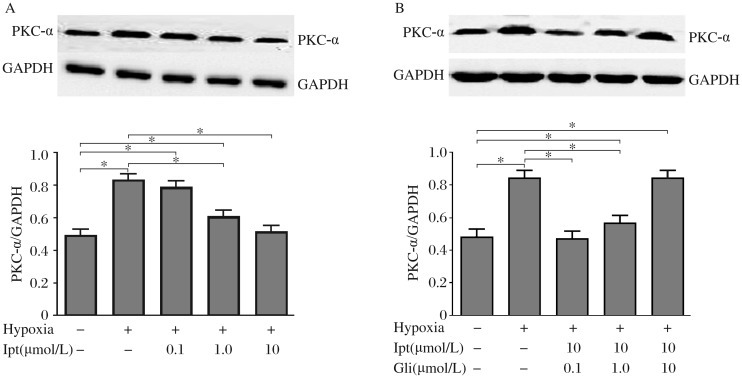
Effect of iptakalim (Ipt) with or without glibenclamide (Gli) on the upregulation of PKC-α expression in human PASMCs induced by hypoxia. A: The human PASMCs were treated with hypoxia for 24 h with or without iptakalim at 0.1-10 µmol/L and iptakalim dose-dependently decreased the expression of PKC-α in human PASMCs induced by hypoxia. B: Pretreatment with glibenclamide at 0.1-10 µmol/L 30 min before iptakalim treatment (10 µmol/L) dose-dependently blocked the downregulation effect of iptakalim. Each value is the mean±SD of 3 independent determinations in each group. *P < 0.05.

***Fig. 6*** showed that iptakalim inhibited the proliferation of PASMCs in a dose-depandent wanner, and the effect of inhibition could be reversed by gliben clamide. our results confirmed that hypoxia increases the expression of PKC-α, and human PASMCs treated with iptakalim (0.1-10 µmol/L) for 24 h decreased PKC-α expression dose-dependently (***Fig. 6A***). glibenclamide abolished the effect of iptakalim on the expression of PKC-α in human PASMCs activated by hypoxia in a dose-dependent manner (***Fig. 6B***). Moreover, glibenclamide (10 µmol/L) completely reversed the decreases in the expression of PKC-α induced by iptakalim. These results demonstrated that hypoxia activated PKC-α and promoted human PASMC proliferation, and iptakalim inhibited the expression of PKC-α by opening K_ATP_ channel.

## DISCUSSION

Our results from both *in vivo* and *in vitro* studies demonstrated that hypoxia increased the expression of PKC-α in PASMCs and promoted PASMC proliferation, which could be prevented by iptakalim. We also found that iptakalim prevented hypoxia–induced pulmonary hypertension and vascular remodeling in rats, which may be due to suppressing the expression of PKC-α in PASMCs. Furthermore, this study showed that the inhibitory effects of iptakalim on PKC-α were completely abolished by glibenclamide, which indicated that the effects of iptakalim on overexpression of PKC-α and pulmonary hypertension induced by hypoxia is associated with the activation of K_ATP_ channels.

Hypoxic vasoconstriction and hypoxia-induced pulmonary vascular remodeling characterized by proliferation and hypertrophy of smooth muscle cells are the key pathophysiological features of hypoxic pulmonary hypertension[28],[29]. In the present study, we observed that hypoxia led to the increased proliferation of PASMCs *in vivo* and *in vitro*, which is in accordance with previous studies[7],[30]. The mechanism of hypoxic pulmonary vascular remodeling is highly complex, and there are still no truly effective drugs for treatments[5]; therefore, we need to search for newer and more effective therapeutic interventions.

PKC-α, a classical Ca^2+^-dependent PKC isotype, plays an important role in smooth muscle cell contraction as well as in the proliferation and apoptotic response of several different cell types[31]. PKC-α is regarded as a key target in the future treatment and management of cardiovascular dysfunction, arterial thrombosis and cancer[11]. Some studies have shown that PKC-α could induce the proliferation of PASMCs, and play important roles in mediating vascular remodeling in the formation of pulmonary hypertension[15],[32].

We observed that the expression of PKC-α increased obviously at the protein level in pulmonary arterioles of chronic hypoxic rats, and PKC-α overexpression was positively correlated with the increase in mPAP and RV/(LV+S) and pulmonary morphological changes after exposure to chronic hypoxia comparing with the control group. In our *in vitro* study, we also found that hypoxia-induced PASMC proliferation was associated with PKC-α overexpression in the hypoxia group compared with the control group, which is in accordance with the results of Dempsey *et al*.[9],[14]. Thus, our results confirmed that hypoxia promotes PASMC proliferation, pulmonary vascular remodeling and pulmonary hypertension, which may be activated by the PKC-α pathway.

The K_ATP_ channels are widely distributed in many tissues and cell types including vascular smooth muscle cells, pancreatic β-cells, neurons, skeletal muscle cells, and cardiac myocytes[33]. Like K_ATP_ channels in other tissues, vascular smooth muscle K_ATP_ channels are now thought to play a vital role as mediators of the response of vascular smooth muscle to a variety of pharmacological and endogenous vasodilators, as well as to changes in metabolic activity that can directly influence blood flow in various tissues[19]. Previous studies have demonstrated that cellular signaling pathways involving serine/threonine kinases, such as cAMP-dependent protein kinase A (PKA) and Ca^2+^ and PKC play a vital role in the amplitude of whole-cell K_ATP_ currents of isolated vascular smooth muscle cells[19],[34]. Activation of PKC-α inhibits potassium current leads to PASMC depolarization and increase of [Ca^2+^]_i_, which may lead to PASMC contraction and proliferation and pulmonary vascular remodeling [14].

It is well demonstrated that K^+^ channel dysfunction plays an important role in the development of pulmonary hypertension. K_ATP_ channel also is involved in hypoxic pulmonary vasoconstriction[35],[36] and proliferation of PASMCs and pulmonary vascular remodeling[23],[37]. It has also been confirmed that opening of K_ATP_ channel by cromakalim attenuated the pulmonary vasoconstrictory effect of PKC activation[27]. With their multiple pulmonary hypertension-related physiological functions, K_ATP_ channels represent promising drug targets[20],[38]. Iptakalim, 2, 3-dimethyl-N-(1-methylethyl)-2-butanamine hydrochloride, has been established as a novel selective K_ATP_ opener with preferential activation of the SUR2B/Kir6.1 subtypes of K_ATP_[22] and shown to be a promising drug for treatment of cardiovascular disease. Our previous studies have shown that iptakalim has an inhibitory effect on [Ca^2+^]_i_ increase and proliferation of PASMCs induced by ET-1 *via* activating K_ATP_ channels[24],[25]. Moreover, chronic treatment with iptakalim attenuates vascular remodeling and control pulmonary hypertension in rats induced by hypoxia[23]. Recently, Wang *et al*.[39] and Long *et al*.[40] demonstrated that iptakalim protects against endothelial dysfunction through activating K_ATP_ channels in endothelial cells. So, iptakalim is a promising drug for treatment of pulmonary hypertension.

In the present study, we showed that the expression of PKC-α in PASMCs under hypoxia was higher than that of the control group and those treated with iptakalim, indicating that iptakalim could inhibit hypoxia-induced PKC-α activity in PASMCs. These results suggested that iptakalim alleviated hypoxia-induced pulmonary vascular remodeling contributed to the inhibition of PASMC proliferation through the PKC signaling pathway. Possible mechanism for these may be that iptakalim activates K_ATP_ channel, leading to K^+^ efflux and promoting PASMC membrane hyperpolarization, which inhibits Ca^2+^ influx, thereby decreasing [Ca^2+^]_i_ in PASMC. while PKC-α is activated by a variety of stimuli in a Ca^2+^-dependent manner[31]. Therefore, iptakalim may decrease the expression of PKC-α and proliferation of PASMCs by reducing [Ca^2+^]_i_ in PASMCs. Iptakalim may also inhibit abnormal elevation of PKC-α *via* correcting the dysfunction of translocation of PKC-α induced by hypoxia.

Therefore, the results of this study could have significant clinical implications and may aid in developing treatments for pulmonary hypertension by providing novel targets of iptakalim for therapeutic intervention.
